# Construct, Face, and Predictive Validity of Parkinson’s Disease Rodent Models

**DOI:** 10.3390/ijms25168971

**Published:** 2024-08-17

**Authors:** Rayanne Poletti Guimarães, Maria Clara Souza de Resende, Miguel Mesquita Tavares, Caio Belardinelli de Azevedo, Miguel Cesar Merino Ruiz, Márcia Renata Mortari

**Affiliations:** 1 Neuropharma Lab, Department of Physiological Sciences, Institute of Biological Sciences, University of Brasília, Brasília 70910-900, Brazil; rayannepg@gmail.com (R.P.G.); clarasdr11@gmail.com (M.C.S.d.R.); migmesquita49@hotmail.com (M.M.T.); caiobelardinelli@gmail.com (C.B.d.A.); miguelcmr@gmail.com (M.C.M.R.); 2 Neurological Rehabilitation Unit, Sarah Network of Rehabilitation Hospitals, Brasília 70335-901, Brazil

**Keywords:** Parkinson’s disease, animal models, construct validity, face validity, predictive validity, experimental medicine, translational research

## Abstract

Parkinson’s disease (PD) is the second most common neurodegenerative disease globally. Current drugs only alleviate symptoms without halting disease progression, making rodent models essential for researching new therapies and understanding the disease better. However, selecting the right model is challenging due to the numerous models and protocols available. Key factors in model selection include construct, face, and predictive validity. Construct validity ensures the model replicates pathological changes seen in human PD, focusing on dopaminergic neurodegeneration and a-synuclein aggregation. Face validity ensures the model’s symptoms mirror those in humans, primarily reproducing motor and non-motor symptoms. Predictive validity assesses if treatment responses in animals will reflect those in humans, typically involving classical pharmacotherapies and surgical procedures. This review highlights the primary characteristics of PD and how these characteristics are validated experimentally according to the three criteria. Additionally, it serves as a valuable tool for researchers in selecting the most appropriate animal model based on established validation criteria.

## 1. Introduction

Parkinson’s disease (PD) is the second most common neurodegenerative disease and the fastest-growing neurological disorder in the world [1]. The main PD pathophysiological characteristics are chronic progressive neurodegeneration of dopaminergic neurons in the substantia nigra pars compacta (SNpc) and aggregation of protein plaques containing a-synuclein and ubiquitin, known as Lewy bodies (LB) [2]. Once dopaminergic neurons of SNpc provide dopamine (DA) to the striatum—a key structure in motor control function—the degeneration of SNpc neurons results in a striatal dopaminergic deficit, leading to the main motor symptoms of the disease: akinesia (difficulty in the onset of movements), bradykinesia (slowness of movements), muscle rigidity, and resting tremor [3]. Despite the use of symptomatic treatments, there are currently no neuroprotective drugs available to stop or prevent disease progression [4]. Also, the cause of PD and all the pathophysiological mechanisms involved are still unclear. Some of the main studied theories about the etiology involve genetic mutations (23 genes have been linked with PD); environmental factors (exposure to pesticides, herbicides, and heavy metals) [5]; and mitochondrial dysfunction (some genes related to familial PD regulate mitochondrial functions, and mutation in these genes leads to mitochondrial dysfunction) [6]. Therefore, preclinical research and the use of animal models are fundamental for clarifying the pathological mechanisms involved in the onset of disease, discovering new therapies, and achieving early diagnosis.

No animal model exactly simulates PD with all pathophysiological mechanisms, symptoms, and treatments seen in humans—since these factors are not all clear yet and all animal models have their limitations. Therefore, the correct and careful choice of the animal model to be used is essential for the success of the study [7]. Rodent models are the most classical animal models used for PD research, due to their ease of handling, small size, and anatomy that is similar to humans [8]. In the 2000s, the most common rodent models of PD are neurotoxin-based models and genetic models [8,9]. In addition, models that combine neurotoxin administration and genetic manipulation have grown in recent years as a way to fill the gaps in each model [8,10].

However, when researchers seek to identify the ideal animal model, two questions often arise: how are these animal models validated and what simulations can each model perform? Indeed, there are three main criteria for validation of animal models of human mental disorders: (i) construct validity, in which the animal model must have a theoretical basis of the psychopathology of the disease; (ii) apparent validity, similarity of the human pathology in the animal model; and (iii) predictive validity, in which the animal model must respond to treatments in a similar way to humans [11]. It is essential to consider these criteria when selecting a suitable animal model.

This review aims to examine the three validities of the primary rodent models used to simulate the main characteristics of PD, while also providing a detailed analysis of the key features of each model, exploring their strengths and limitations. Also, we provide an updated resource to help researchers choose the animal model that best corresponds to their interests.

## 2. Construct Validity

Construct validity is a term used to suggest that a procedure is based on a previously known solid theory. Regarding animal models, it applies to the possibility of simulating pathological theories of human disease in non-human species [11]. In other words, when an animal model has construct validity, it means that the model simulates at least one pathological change observed in human disease, without necessarily simulating the outcome of these changes—as signs observed in patients. Construct validity requires homology and demonstration that the characteristic is fundamental for the disease, rather than epiphenomenon superficial similarity, which can be assessed through apparent validity [11].

Thus, the applicability of the term requires that the pathological features of the disease have been studied previously in humans to be applied to animal models. Therefore, not all neurological diseases have animal models with construct validity; some are still in the process of discovering their pathological basis. Also, animal models will not have construct validity for all the pathological features of the disease, because many of these features may still be being discovered.

**Figure 1:**
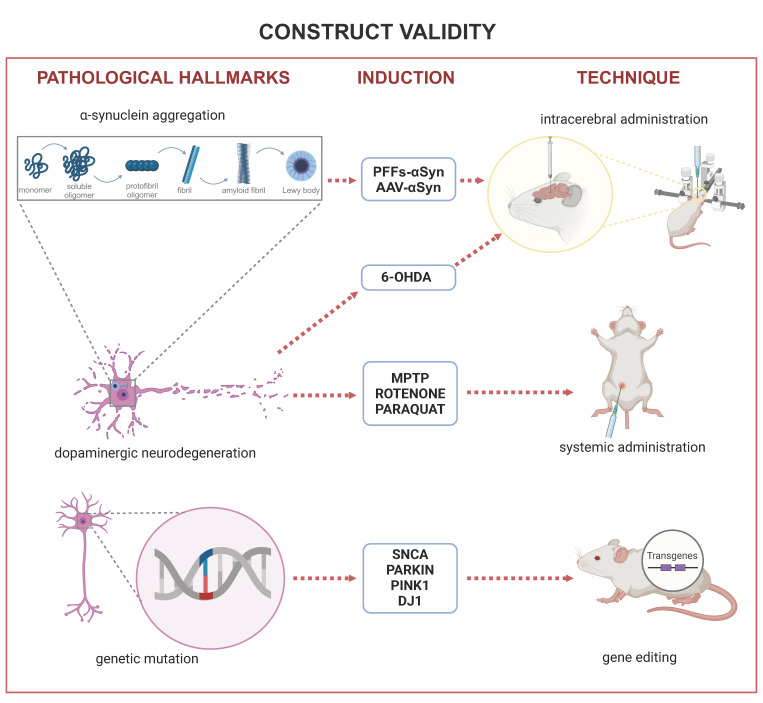
Construct validity in rodent models of Parkinson’s disease: key pathological hallmarks simulated in models, primary induction methods, and techniques used for induction.

In this session, we will discuss the construct validity of rodent models of PD that simulate its three main pathological features: (i) chronic progressive neurodegeneration of the dopaminergic neurons of the SNpc, (ii) presence of protein aggregates containing a-synuclein, simulating Lewy bodies, and (iii) mutations of PD-related genes: SNCA, Parkin, Pink-1, and DJ-1. Summarized information can be found in Table 1 and Figure 1.

### 2.1. Degeneration of Dopaminergic Neurons

The chronic progressive degeneration of DA neurons in the SNpc is one of the main hallmarks of PD [2]. Since dopaminergic neurons in the SNpc project their axons to the striatum via the nigrostriatal pathway, neurodegeneration of neurons in the SNpc leads to a dopaminergic deficit in the striatum. The absence of DA in the striatum entails an imbalance in the activities of the direct and indirect pathways of motor control, which is responsible for the main motor symptoms of PD [3]. Therefore, dopaminergic neuron degeneration is very well established as a hallmark of PD and used in many animal models to mimic the disease. Neurotoxin-based models are the most commonly used to mimic the neurodegeneration of dopaminergic neurons, mainly 6-hydroxydopamine (6-OHDA), 1-methyl-4-phenyl-1,2,3,6-tetrahydropyridine (MPTP), and some pesticides [8].

The 6-OHDA neurotoxin model was described for the first time in 1968 [37]. 6-OHDA is a highly oxidizable DA analog molecule uptake by dopamine transporter (DAT) into dopaminergic neurons, causing highly selective lesions [38]. Once inside neurons, 6-OHDA initiates an auto-oxidation process, producing hydrogen peroxide, superoxide, and hydroxyl radicals, resulting in oxidative stress and dysfunction of complex I of the mitochondrial respiratory chain, leading to cell death [16]. Mitochondrial dysfunction and complex I inhibition are considered key elements in PD pathogenesis [39], evidenced by post-mortem findings in SNpc, skeletal muscle, and platelets of PD patients [40]. These elements and features correlate with the mechanisms of action of the main neurotoxins that induce neurodegeneration in rodent models of PD.

As 6-OHDA does not cross the blood–brain barrier (BBB), administration occurs intracerebrally—through stereotaxic surgery—in three main brain structures: striatum, SNpc, or medial forebrain bundle (MFB), generating lesions with different characteristics. When administered into the striatum, 6-OHDA destroys the terminal axons of neurons projecting from the SNpc, causing slow retrograde neurodegeneration throughout 1 to 3 weeks [41,42]. Also, the striatum can receive one to four injections of 6-OHDA, achieving 30–75% neuronal death depending on the dose and number of injections administered. In SNpc and MFB, 6-OHDA administration results in significant and rapid injury. Injury begins within 24 h of injection and reaches its maximum DA reduction in the striatum in about 3 to 4 days. Furthermore, neuronal loss can reach more than 90% in a period of up to 5 weeks after 6-OHDA administration [43].

In contrast to 6-OHDA, MPTP is known to easily cross the BBB, because of its lipophilic characteristic. Thus, intracerebral administration is not necessary, as it can be administered only systemically—which in turn can make the process more hazardous for researchers [44,45]. After crossing the BBB, MPTP is converted into the 1-methyl-4-phenylpyridinium ion (MPP+) through the enzymatic action of monoamine oxidase B (MAO-B) in glial cells. MPP+, the toxic metabolite, is then transported into the dopaminergic neurons via DAT, causing a selective lesion, as well as 6-OHDA [16]. Once inside neurons, MPP+ inhibits complex I of the mitochondrial respiratory chain, decreasing ATP generation and increasing the production of reactive oxygen species (ROS), leading to neurodegeneration [16].

In general, rodents are less sensitive to MPTP when compared to primates [8]. Rats are rarely used to model PD with systemic administration of MPTP because the dose required to induce cell degeneration entails a high mortality rate [46]. Thus, some studies used other forms of administration in rats, such as intracerebral administration of MPP+ and intranasal administration of MPTP [47,48,49]. In mice, the MPTP model is more reproducible and is considered easy to handle and affordable [50]. Moreover, other biomarkers can be found in the MPTP mouse model: one study correlated blood biomarkers from PD patients and mice, showing that among 13 blood biomarkers from PD patients, 7 were detected in symptomatic mice and 3 in symptomatic and pre-symptomatic mice, confirming the validity of this model [51].

Also, among the category of neurotoxin-based models, rotenone and paraquat are pesticides frequently used to model PD in rodents to investigate potential environmental hazards, pesticide exposures, and their relation to the disease. Studies investigate the relationship between human exposure to these pesticides and increased risk of PD, but there is still controversy about the conclusion of these studies which some claims are based on insufficient data from epidemiological studies [52]. Both pesticides can cross BBB, due to its lipophilic characteristic, and act to degenerate dopaminergic neurons in the SNpc through oxidative stress. Rotenone inhibits complex I of the mitochondrial respiratory chain, just like 6-OHDA and MPTP [53,54]. Paraquat—in contrast to the other neurotoxins—acts by impairing the redox recycling of glutathione and thioredoxin, damaging the intracellular antioxidant system [16,55,56].

However, although neurotoxin-based models were the most used in the 2000s [8], most of the new treatments studied in these models do not translate to clinical studies due to their limitations, which raises concerns regarding their reliability. One of the main limitations discussed about the toxin-based models is that it does not simulate the progressive time course of dopaminergic neuron degeneration and, during the progression of the disease, some compensatory changes may appear in the patient’s brains, a feature that does not occur in the acute toxin model [57]. Furthermore, additional limitations include the absence of LB-like inclusions and the lack of neuron specificity—as these models may affect brain regions unrelated to PD [58] or they may not fully capture the involvement of other pathways associated with the development of non-motor symptoms in PD. Additionally, concerning the reproducibility of the models, it is important to consider the sex of the animals, since—as in the human disease—there is some evidence of sexual dimorphism in PD models. In the MPTP and 6-OHDA models, male rats appear to have a greater loss of DA neurons than females [59,60,61].

Besides neurotoxin-based models, some genetic or a-synuclein-induced models can also cause degeneration of dopaminergic neurons in SNpc, although this is not their main hallmark, since the use of these models often aims at studying the function of specific genes, a-synuclein, or the interaction between these two subjects. Among the gene mutations that have been shown to cause degeneration in dopaminergic neurons is the I93M mutation in ubiquitin carboxyl-terminal hydrolase L1 (UCH-L1), found in a family with hereditary PD [62]. Transgenic mice high-expressing I93M showed a significant reduction in dopaminergic neurons in SNpc and the DA content in the striatum [63]. Also, deficiency in the transcription factors nuclear receptor-related 1 protein (Nurr1), engrailed 1 (EN1), pituitary homeobox 3 (Pitx3), sonic hedgehog (SHH), and c-Rel—which play an important role in the development and maintenance of dopaminergic neurons of the nigrostriatal pathway—have been shown to cause significant degeneration in dopaminergic neurons of the SNpc [64]. Induction of a-synuclein overexpression through the adeno-associated virus (AAV) showed dopaminergic neurodegeneration after 8 weeks post-injections [65]. Also, the injection of synthetic a-synuclein fibrils into the striatum of non-transgenic mice showed a significant loss of DA neurons in the SNpc at 180 days post-injection [28].

Moreover, it is crucial to highlight that models requiring intracerebral administration may be susceptible to administration errors by researchers lacking extensive training in stereotactic surgeries. Typically, several techniques are commonly utilized in confirming the degeneration of dopaminergic neurons within these models, including immunohistochemistry/immunofluorescence for the staining of tyrosine hydroxylase (TH), an enzyme present in dopaminergic neurons involved in L-DOPA synthesis, and amphetamine/apomorphine-induced rotation test, a behavioral test used to predict cell loss through quantification of ipsilateral rotation in unilaterally lesioned rodents [7].

### 2.2. Lewy Bodies

Lewy bodies (LBs) are cytoplasmic deposits made up of various proteins, including ubiquitin, tau, and parkin, with most of the proteins present represented by phosphorylated a-synuclein [66]. a-synuclein is a 140 amino acid residue protein, abundant in the brain and located mainly in pre-synaptic regions [67]. The term synuclein refers to proteins that are located close to nuclear and synaptic membranes [68]. a-synuclein is encoded by the SNCA gene and its functions have not yet been fully clarified, but studies indicate that under normal physiological conditions, a-synuclein plays a role in modulating the release of neurotransmitters from synaptic vesicles into the extracellular environment [69], and its over-expression is capable of inhibiting the release of neurotransmitters in neuron cultures [68]. In a healthy context, a-synuclein is found in the form of monomers or a-helix in the brain, and can be converted to β-sheets—its pathological form—through the recruitment of monomers that will trigger the formation of amyloid fibrils, the main components of LBs [70].

The presence of a-synuclein is one of the main biomarkers of PD [71]. However, although deregulated protein aggregation is recognized as a common feature of neurodegenerative diseases, little is known about how a-synuclein aggregates contribute to neurodegeneration and the development of motor and non-motor symptoms in PD. Most animal models have for years focused on reproducing neurodegeneration through neurotoxins which, for the most part, do not generate a-synuclein aggregates and do not contribute to understanding the pathogenicity of the protein [8,72]. A rodent model that could reproduce the aggregation of a-synuclein together with a progressive loss of DA neurons in the SNpc would be the most complete model in terms of construct validity since it would simulate the two most studied characteristics of the disease. The use of animal models with these two characteristics would help to boost the translational aspect between preclinical research and clinical research, reducing, for example, the high number of new treatments that fail in clinical trials.

However, the generation of PD models that reproduce the presence of a-synuclein aggregates has been steadily increasing. These models are mainly based on genetic modifications of rodents (see next section), AAV-aSyn based models, or infusion of a-synuclein-preformed fibrils (PFFs) [73]. PFFs and AAV-aSyn have been used mainly to study the early stages and the progression of the diseases [73]. Models based on the infusion of a-synuclein are characterized by the injection of a virus containing a transgene of a-synuclein under the presence of a promoter, leading to moderate cell loss after 22 weeks and behavioral deficits after 4–8 weeks after injection in SN [74]. PFFs are formed in vitro [75] and infused into the brain (striatum, cortex, hippocampus, SN, etc.), olfactory bulb, and gastrointestinal system, among others. Studies suggest that exogenous PFFs influence the aggregation of endogenous a-synuclein and the formation of plaques similar to LBs, possibly causing synaptic dysfunction, and long-term neuronal death [76], which can take up to 6 months to appear, as well as the development of late motor signs, which in some cases may also not appear [77]. Furthermore, regarding sex differences, male mice appear to be more susceptible to a-synuclein toxicity when compared to female mice [78].

Although most of the studies in animal models induced by neurotoxins, herbicides, and pesticides do not show the presence of a-synuclein aggregation, some evidence suggests that a-synuclein aggregates can also be found in models with neurotoxins such as MPTP, depending on the type of treatment. A study showed that treatment with sub-acute doses of MPTP was able to generate a-synuclein inclusions [79], but treatment with acute doses of MPTP did not show the presence of aggregates [80]. On the other hand, the 6-OHDA infusion model was unable to simulate the LBs in the animals’ brains [79]. The combined treatment of paraquat (herbicide) and maneb (fungicide) is capable of causing a-synuclein aggregates in rodents after 6 weeks of administration of two weekly doses [81]. Moreover, other studies suggest that Paraquat, and not Maneb, is responsible for generating the aggregates [82]. The PD model induced by the pesticide Rotenone also showed the presence of a-synuclein aggregates after 5 consecutive weeks of treatment [83].

### 2.3. Gene Mutations

Genetic models have proven to be a valuable tool for understanding the pathogenesis of PD. Several genes have been identified as responsible for different forms of PD, including mutations in the α-synuclein gene, which have been found in families with Parkinsonism [84]. Furthermore, the study of genetic risk factors has been instrumental in identifying the causes of idiopathic PD, as demonstrated in a recent review of genetic risk factors in PD [RP1] [85]. Genes such as DJ-1 and Parkin, for example, have been associated with recessive forms of juvenile Parkinsonism [86]. Additionally, the PINK1 gene has been identified as responsible for hereditary forms of early-onset Parkinsonism [87]. All of these genetic models have contributed to elucidating the underlying pathogenic mechanisms of PD, facilitating the development of new therapies and treatments. Some of these models present crucial characteristics that allow the study of specific features of PD, such as the onset of the disease. Some models can mimic the characteristics of the disease in humans, such as an early or late onset, depending on the affected gene. A study that investigated the relationship between risk genes and the onset of the disease demonstrated a low frequency, but strong relationship between these genes and the onset of PD [88]. This may indicate that genetic models are important for a better understanding of the impact of these genes on the onset of the disease.

#### 2.3.1. SNCA

a-Synuclein is a widely studied neuronal protein due to its association with PD [86]. The SNCA genetic model, which involves the expression of human a-synuclein driven by the TH promoter in transgenic mice, has been used to investigate the pathophysiological mechanisms of the disease [89]. Research with SNAC transgenic mouse models has shown that the presence of a-synuclein is not associated with the loss of dopaminergic neurons, despite the presence of a-synuclein aggregates in nerve cells and behavioral manifestations similar to those observed in PD patients [89]. However, the expression of a-synuclein in mice without the a-synuclein gene resulted in a reduction in the size of dopaminergic neurons and alterations in their dendritic morphology [90]. Furthermore, a-synuclein can affect neurotransmission through its interaction with synaptic vesicles. Studies have shown that calcium binding to the C-terminal of a-synuclein modulates the interaction of synaptic vesicles, leading to alterations in neurotransmitter release [34]. However, other studies indicate that pathogenic mutations in a-synuclein affect the conformational balance of the protein, resulting in increased formation of toxic protein aggregates that can lead to neuronal death [91,92,93].

Some point mutations (A30P, E46K, H50Q, A53T) and copy number mutations in the a-synuclein gene are commonly related to an autosomal dominant type of PD in humans. These point mutations can exhibit distinct clinical and functional characteristics, each uniquely contributing to the phenotypic variability observed in PD [94]. The A53T mutation is typically associated with early-onset disease, rapid progression, and a broad spectrum of non-motor symptoms, including cognitive and psychiatric disorders. Conversely, the A30P mutation is linked to later disease onset with more limited non-motor manifestations, primarily cognitive impairment [95]. The E46K mutation is known for causing severe parkinsonism, often accompanied by dementia, dysautonomia, and psychiatric symptoms, with recent findings indicating variability in disease severity and progression within families [95]. Lastly, the H50Q mutation, though rarer, accelerates α-synuclein aggregation and increases its toxicity, leading to cognitive decline and other non-motor symptoms over time [96]. These point mutations can be explored to develop new animal models of PD, that are already being used.

A study was conducted with two transgenic animal models, each carrying one of the mutations A53T or A30P, both of which exhibited abnormalities in the enteric nervous system [97]. However, only the A53T model demonstrated a motor deficit, despite the absence of signs of progressive neurodegeneration. Additionally, in another study, mice with the A30P mutation exhibited reduced levels of dopamine in their brains, which helped demonstrate that a single point mutation is sufficient to induce age-related decline in motor functions, which is a phenotype characteristic of PD [98]. These animal models serve as valuable tools for elucidating the earliest abnormalities in neuronal function and subsequent pathogenic processes leading to nervous system degeneration in PD [99]. The point mutation E46K is associated with the familial and hereditary form of PD, as well as LB dementia [100]. A mouse model with overexpression of the E46K point mutation suggests that the way αSyn functions in intact synaptosomes impacts the shape and function of synaptic vesicles. This animal model exhibits disruptions in the vesicle transport process promoted by a-synuclein, leading to neuronal death and generating motor signs similar to those observed in PD, such as resting tremor, which is reversible with L-DOPA treatments [101]. Some researchers demonstrated that the H50Q point mutation can accelerate a-synuclein aggregation, secretion, and toxicity in cell-based models [96], but until the current moment, no assays have been described with mouse models carrying the H50Q point mutation.

#### 2.3.2. PARKIN

The protein encoded by the parkin gene (PKRN) is an E3 ubiquitin ligase, called parkin, responsible for the ubiquitination and degradation of damaged or misfolded proteins through the proteasomal pathway. Its function is essential for maintaining cellular homeostasis, particularly concerning mitochondria and cellular energy production [102]. The PKRN is one of the genes associated with the autosomal recessive juvenile form of PD [103] and plays an important role in regulating mitochondrial homeostasis and the elimination of damaged proteins [104]. It is believed that the loss of parkin protein function, the elimination of dysfunctional mitochondria, and the impairment of damaged proteins leads to an increase in ROS, promoting exacerbated oxidative stress in the cell environment, and leading to neuronal death and disease progression [105]. A study in a genetic mouse model with parkin deletion revealed increased DA concentration in the striatum, reduced synaptic excitability, and behavioral deficits, such as motor impairments. However, the animals did not show a significant loss of dopaminergic neurons in the SN, suggesting a non-essential role of parkin in the survival of these neurons [103].

#### 2.3.3. PINK1

The PINK1 gene encodes a protein with a crucial role in maintaining mitochondrial homeostasis. Studies have demonstrated that the PINK1 protein is selectively stabilized in defective mitochondria, where it recruits the E3 ubiquitin ligase Parkin and activates it, triggering the selective degradation of these mitochondria through the process of mitophagy [106]. Additionally, studies using animal models with loss of function of the PINK1 gene have shown that the absence of this protein leads to alterations in the activity of mitochondrial respiratory chain complex I. Loss of PINK1 results in uncoupling of ubiquinone NdufA10, a subunit of complex I, leading to increased production of reactive oxygen species and compromised mitochondrial function [107]. The PINK1 protein is also capable of phosphorylating the mitochondrial chaperone TRAP1, protecting it against oxidative stress [108], where the dysregulation of the gene leads to organelle dysfunction. Overall, studies indicate that the PINK1 protein plays an important role in maintaining mitochondrial homeostasis, acting both in the identification and signaling of defective mitochondria for elimination through mitophagy, as well as in protection against oxidative stress [106,107,108].

The animal model with PINK1 gene deletion exhibits pathophysiological features that mimic some characteristics of PD [109]. Experiments conducted in mice with loss of function of the PINK1 gene have revealed a reduction in the activity of mitochondrial respiratory chain complex I, along with an increase in the generation of reactive oxygen species and mitochondrial dysfunction. These alterations are similar to those observed in PD patients and may increase susceptibility to oxidative damage and induction of apoptotic processes [109,110]. Other studies using transgenic models with KO of PINK1 demonstrate the relation in PINK1 deletion with age progression and show that young adult PINK1 KO rats exhibited hyperkinetic behavior with elevated DA and TH levels in the SN, but not motor deficits, and suggest compensatory mechanisms to preserve locomotor function in response to deficient PINK1, which decline with aging and the onset of motor decline [111]. Another study with PINK1 knock-down mice corroborates the findings, demonstrating that PINK1-deficient mice exhibit non-motor signs that occur in the early stages of PD, such as olfactory dysfunction [112], and is in parallel with the idea that animal models deficient in PINK1 do not present early neurodegeneration due to a compensatory mechanism. Therefore, genetic models of PD deficiency for PINK1 are useful models for studying the initial and non-motor signs of PD, facilitating the study of the development of the disease [113].

#### 2.3.4. DJ-1

The DJ-1 gene, also known as PARK7, is a human gene that encodes a protein that plays an important role in the survival and function of dopaminergic neurons. Mutations and loss of function of the DJ-1 gene are associated with recessive forms of familial parkinsonism [85]. Studies suggest that the DJ-1 protein has antioxidant activity and helps protect against the oxidative environment of dopaminergic neurons [85]. A study with a genetic model of DJ-1 deficient mice showed motor deficits in the animals at 5 months of age and did not exhibit significant loss of dopaminergic neurons at 6 or 11 months of age [114]. This differs from human PD patients, who typically develop motor symptoms only after losing 50 to 60% of dopaminergic neurons [115]. However, DJ-1 deficient mice exhibited hypokinesia in open field tests, indicating that DJ-1 deficiency can lead to motor dysfunction even in the absence of neurodegeneration in the SN [116,117]. Furthermore, it has been demonstrated that mice with DJ-1 gene deletion are more susceptible to insults with MPTP and rotenone, suggesting that the DJ-1 protein plays a neuroprotective role in the neural system [118].

#### 2.3.5. Genetic Variability and Clinical Trial

The significant genetic variability of the disease, coupled with the existence of as-yet undescribed genes that may be linked to PD, introduces a potential source of bias in research. This means that observed results could stem from differences in the genetic profiles of patients in a given study rather than from the therapeutic intervention itself. Moreover, genetic variability not only influences the disease phenotype but also the metabolism of drugs, further complicating clinical trial outcomes. Variants in genes related to drug metabolism can alter how patients respond to treatment, leading to variable efficacy and adverse effects across the trial population [119]. This genetic heterogeneity is even more problematic in small samples, where the effects of the imbalance in genetic distribution are more pronounced, for example in studies that use families with a low number of individuals for the study.

To address these challenges, strategies such as genetic stratification and controlled randomization have been proposed to mitigate the impact of genetic variability on clinical trial outcomes. By genotyping participants before the trial and ensuring a balanced distribution of known genetic variants across treatment and placebo arms, researchers can reduce the likelihood of bias due to genetic differences. This approach not only enhances the validity of the trial results but also increases the statistical power of the study, allowing for more accurate detection of true drug effects [120].

## 3. Face Validity

Face validity was the first criterion established for the validation of animal models. McKinney and Bunney (1969) proposed the general guidelines for this assessment, seeking to integrate more objectivity in animal studies. Face validity can be considered the most traditional form of validating animal models since it was the first one ever proposed [11]. This form of analysis was meant to make tracking behavior changes more objective and systematic. In this regard, the assessment of a set of observations should be impartial in the sense that independent evaluators should reach the same objective conclusions. Therefore, the behavior of the animals analyzed had to fall under the criteria already established for it to be valid in the context of its study.

**Figure 2:**
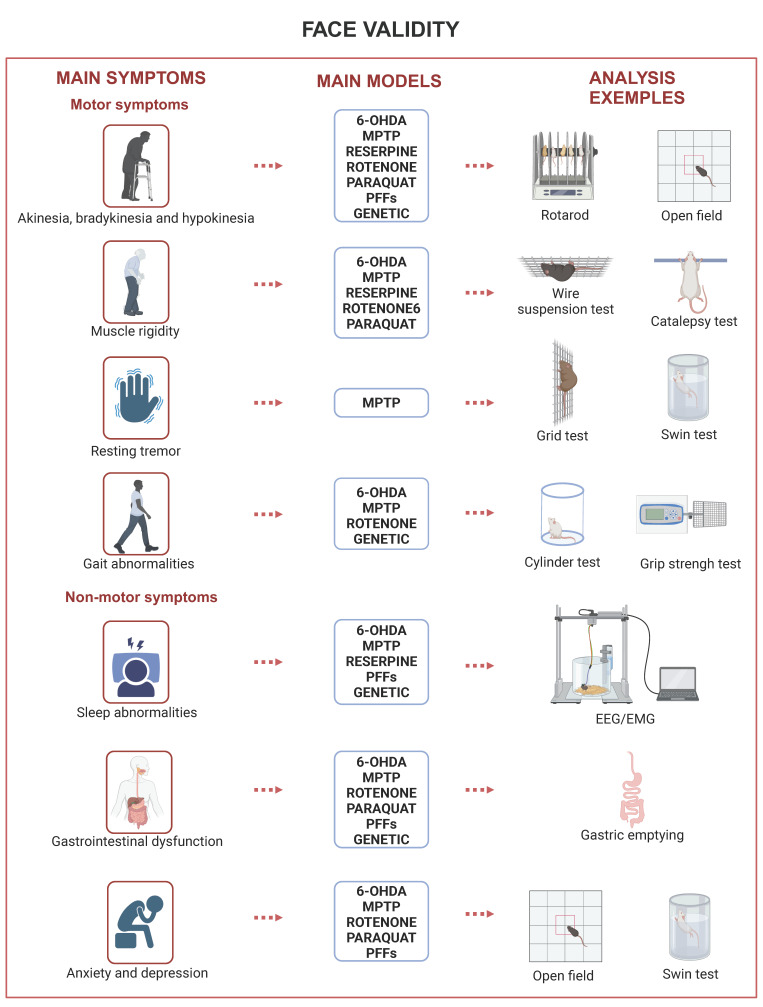
Main symptoms of Parkinson’s disease, primary rodent models capable of simulating it, and tests used for face validity analysis.

Furthermore, this form of validity had to encompass aspects that went beyond behavioral changes. There should be similarities in etiology, symptomatology, biochemical parameters, and treatment [121] for the model to be considered valid. As other validation criteria were proposed, the definition of face validity, whose main pillar remained the similarity in symptoms, was also broadened. The first addition regards the dissimilarities, establishing that there should not be major dissimilarities between the animal model and the condition it intends to model [11]. The second addresses the specificity of the model, emphasizing that the symptoms should correspond to the pathology they intend to model, not to general aspects of different conditions [122]. Summarized information concerning the following sections can be found in Table 2 and Figure 2.

### 3.1. Motor Symptoms

Akinesia, muscular rigidity, resting tremor, and gait abnormalities are some of the cardinal motor symptoms of the disease. These and other symptoms have been associated with the loss of dopaminergic neurons and the depletion of DA in the striatum [3]. The death of neurons in the SNpc leads to a decrease in the levels of DA in the striatum, causing a loss of motor control [3]. Most of the models developed for PD manifest one or more motor symptoms. Since they are such a cardinal symptom of PD models, many articles do not differentiate between them, simply stating that there are motor dysfunctions in the model they have worked on.

When it comes to sex differences, males tend to exhibit more motor symptoms than females [78,178]. MPTP and 6-OHDA seem to cause higher motor impairment in male rats compared to females [150,179,180]. Also, studies with reserpine have shown that female rats are more resistant to motor dysfunctions than males. In that sense, their motor impairments are delayed or prevented when compared to males [181].

#### 3.1.1. Akinesia, Bradykinesia, and Hypokinesia

Akinesia can be defined as difficulty in the onset of movements and lack of spontaneous movement. Bradykinesia can be defined as slowness of movements, and hypokinesia is a reduced amplitude of movement. These symptoms often manifest themselves together and can be assessed through many of the same tests. To avoid redundancy, they were grouped in this topic.

These symptoms can be validated through most animal models, since inducing these kinds of motor symptoms and dopaminergic loss in rodent models is one of the most thoroughly reproduced aspects of the disease. Usually, akinesia, bradykinesia, and hypokinesia can be assessed by using motor coordination tests. The rotarod test is an example, it is employed in several PD animal models because it allows for the observation of motor coordination and stamina. It assesses motor skills by placing animals on a spinning cylinder that is accelerating and measuring their latency to fall [14]. Open field is also one of the main tests employed to assess this kind of behavior across most models. It is used in behavioral testing to track the animal’s locomotion patterns and rearing since it is placed in a circular open field arena for free exploration for a specific amount of time [151] to assess spontaneous locomotion [18].

When it comes to models, the 6-OHDA model is one of the most established PD models and involves several motor symptoms, akinesia being one of them [58]. Tests such as the tail suspension test (TST)—rodents are suspended by their tails with tape and their escape-oriented behaviors are quantified, tail suspension swing test (TSST)—monitoring the temporal evolution of the swings [126] can be used to assess the emergence of motor deficits. The pole test is also an established test for assessing locomotion deficits. The mouse is placed with its head up on the top of a vertical pole and the time for it to reach the floor is measured to analyze locomotor activity [125].

Similarly, the balance beam test, employed in the model of MPTP, analyzes motor coordination and balance. Latency to cross the beam and number of foot slips are documented during this test [140]. Different diameter circular beams can be employed. The test usually ends when the animal completes the given test or when the cut-off time is reached. Forelimb akinesia can also be determined by employing the stepping test, originally designed to analyze asymmetric motor deficits in rats [137]. In that sense, it can be seen if the rodents present a reduction in the number of adjusting steps [141]. Horizontal and vertical grid tests and swim tests can also be employed for assessing akinesia, tremor, and rigidity in MPTP and can provide sensitive measurements of motor deficits [143,144]. The pole test can also be employed to assess locomotion deficits in MPTP models [142].

Injection of reserpine—an inhibitor of the vesicular monoamine transporter (VMAT)—causes monoamine depletion and can induce the development of akinesia as well [147]. It can be assessed by using the catalepsy test to analyze immobile posture. The animal’s forepaws are placed on a horizontal bar, and the duration of catalepsy, which is keeping both forepaws on the bar, is measured [151].

Rotenone models exhibit motor coordination deficits (akinesia, bradykinesia, hypokinesia), which can be observed as an increase in the number of falls on the rotarod, an increase in immobility, and a decrease in climbing on the forced swimming test, as well as alterations in the pole test and the catalepsy test [157].

A decline in locomotor activity can also be observed in Paraquat models, affecting akinesia, hypokinesia, and bradykinesia. To assess these symptoms, the catalepsy test is employed to assess bradykinesia. Postural reflexes can be analyzed by employing the inclined plane test to assess whether the animal can keep his body in the inclined plane. The swimming test assesses complex movements, where the animal is placed in a water-filled recipient and his performance is evaluated [161].

The PFF model’s motor impairments might become apparent with the administration of higher doses of PFFs or at later time points [157]. They can be assessed by the wire hang test (the animal is positioned on the cage top, which is then inverted and its latency to fall is measured), tail suspension test (TST) [28], muscular strength test (rodents are held by their tail and grasp the wire grid of the grip strength meter with their forepaws, and are then gently pulled backward by their tail until grid release), elevated plus maze test (distance traveled between maze arms), forced swim test, and Y-maze test (alternation of entries into all three arms of the maze) [165].

Genetic models are a broad spectrum and illustrate the complexity of PD models when it comes to genetic factors. The vast majority of transgenic models show some kind of motor deficit [147]. Studies with the point mutation mouse model, which introduces genetic mutations similar to the ones present in familial cases of the disease, such as DJ-1, PINK1, Parkin, and α-synuclein, have shown motor symptoms such as bradykinesia, akinesia, and hypokinesia [98]. Viral overexpression of genes, such as α-synuclein, and the disruption of nigrostriatal neurons—through MitoPark and Nurr1 for example—also have an impact on motor function [182]. The animals show an increased number of falls on the rotarod and a decrease in overall motor activity, which can be assessed by the balance beam test [183] and its variations, such as the tapered balance beam test [171]. A tail suspension test (TST), grip strength test, and pole test can also be employed, and the adhesive removal test (time the animal takes to remove the adhesive label on the snout) can be used to assess sensorimotor deficits [172].

#### 3.1.2. Muscle Rigidity

Bilateral intracerebral administration of 6-OHDA can be used to mimic muscle rigidity in rats, and the grasping test [123] or movement-induced reflex electromyographic activity can be used for analysis [124]. As mentioned in the akinesia section, horizontal and vertical grid tests and swim tests can be used to assess akinesia, tremor, and rigidity in MPTP [143,144]. Reserpine models mimic the rigidity observed in clinical features of PD [147]. It can be a model of transient PD symptoms with a degree of reversibility. L-DOPA can partially rescue the effect of reserpine administration. A partial recovery of nigral DA neurons occurs 30 days post-injection, rescuing motor deficits [150]. Studies report that Paraquat exposure affects forelimb performance by causing rigidity [162]. The wire suspension test can be employed to assess this. The test allows the mouse to grasp a horizontal wire and then evaluates its ability to pull its hanged body by flexing its forelimbs. The catalepsy test can also be used to analyze muscle rigidity in the models of MPTP, reserpine, rotenone, and paraquat.

#### 3.1.3. Resting Tremors

When it comes to resting tremors, there are not many models that exhibit this motor symptom. The main model that shows this symptom is the MPTP mode. For this model, horizontal and vertical grid tests and swim tests can be used to analyze akinesia, tremor, and rigidity in MPTP [143,144], as aforementioned.

#### 3.1.4. Gait Abnormalities

Gait abnormalities can be defined as abnormal walking patterns that deviate from normal walking or gait. They can be observed in animal models by analyzing average speed, base of support, cadence, swing speed, stride length, step cycle, duty cycle, stance, and regularity index, among other parameters [140]. Analyzing gait parameters can also help assess other motor symptoms, such as bradykinesia, muscle rigidity, and hypokinesia. The stride length assessment may correlate to muscle rigidity and hypokinesia [141,143].

The following models have been employed to assess gait alterations: 6-OHDA [127,184], MPTP [128,140], rotenone [156], and genetic models [115,173]. One of the main tests applied to analyze gait is the cylinder test, which is used to measure the animal’s spontaneous forelimb use and its asymmetry [133]. The rodent is put in a glass cylinder and the number of times it rears up and touches the cylinder wall is monitored. These wall touches are then scored for the right, left, or both paws [183]. This can be used to evaluate the sensory-motor function.

In genetic models, gait can also be assessed by grip strength, where the animals are held by their tails, gently pulled across two push-pull gauges (one for their forelimb and one for their hindlimb), and the grip force for the forelimbs and hindlimbs is recorded [173]. The ink test can also be employed to analyze gait alterations. In this test, animals are trained to run across a course covered with white paper and into a dark container. Their forefeet are marked before the test to measure their stride width and length [98].

### 3.2. Non-Motor Symptoms

PD treatment models over the years have mostly focused on striatal function and DA replacement therapy [185]. Furthermore, their face validity was heavily based on behavioral motor symptoms. However, researchers have realized the disease is much more than that. Non-motor symptoms are being incorporated into a battery of tests when analyzing behavioral aspects of PD. Therefore, giving PD models more complexity through face validity.

As for non-motor symptoms, depression anxiety, sleep abnormalities, and gastrointestinal (GI) dysfunction are the more common ones. Although they are less talked about, non-motor symptoms of PD are a big part of the disease and affect many patients. GI dysfunction affects more than 70% of PD patients [186], and it has been shown that it can be replicated in mouse models. Sleep abnormalities extending from night-time awakenings, sleep fragmentation, and REM sleep disorder occur in 65–95% of PD patients [134]. Depression and anxiety affect about 40% of the population with PD [136]. In regards to sexual differences in non-motor symptoms, some case studies have shown that women tend to have more depression [187] and anxiety [188] than men. Nevertheless, no study has yet been able to reproduce such behaviors in rodent models.

Non-motor symptoms are harder to incorporate in models, but they have been shown to give great depth to the research. Therefore, understanding and considering them when designing a model is crucial.

#### 3.2.1. Sleep Abnormalities

The most trustworthy way to assess sleep disorders is through electroencephalogram (EEG) and electromyogram (EMG) [189]. They can be used to determine in which phase of the circadian cycle the animals are and evaluate their quality of sleep. Furthermore, tests evaluate sleep behavioral signs of sleep, which can be determined by posture and breathing patterns [190]. The behavioral tests have been shown to correlate with EEG data [191].

Toxin models are a staple in PD studies and sleep abnormalities are no different. Regarding toxins models, the 6-OHDA model replicates PD-related sleep dysfunctions as seen in humans the best, with EEG oscillations being observed. However, the site of the lesion will determine what PD-like signs will be replicated. A study showed a significant difference in sleep duration in 6-OHDA-treated animals, including both NREM and REM [192]. Another study observed a difference in decreased wakefulness and increased NREM sleep during the active period [134].

Although the MPTP model seems to present sleep abnormalities, most studies report it as transitory [193]. A study observed an increase in REM latency in MPTP rats but lasted only 3 days after the lesion [145].

Reserpine can replicate sleep abnormalities, but to a smaller degree than the 6-OHDA model study showed higher REM and NREM in the active phase, while having lower NREM in the inactive phase [152].

Among the genetic animal models for PD, α-synuclein shows the best resemblance to what is observed in humans, although α-synuclein presents lower neuronal loss than neurotoxins. Nevertheless, it does not impact the results since sleep disorders happen in PD’s early stages. A study with the administration of α-synuclein in mice found a higher rate of NREM sleep in the inactive phase, it was also observed a reduction in REM sleep [176].

The PFF model does not have the number of studies for sleep as other models. Disorders can only be seen if the infusion happens in the laterodorsal tegmental nucleus [166], and not in the striatum which is the standard for the model.

Sleep disorders are not studied as much as other aspects of the disease. Therefore, not all models have studies analyzing their effects on the animal’s sleep, for example, models like Paraquat lack studies regarding the topic.

#### 3.2.2. Gastrointestinal Dysfunction

Gastrointestinal issues in PD are a common symptom and many think it can shed light on the disease and increase our understanding. There are many ways to assess GI dysfunctions, measuring urinary excretion of sucralose/lactulose in mice which shows similar results in humans when conducting the same experiment, showing intestinal leak, especially in column [159]. Another test is gastric emptying, which consists of having the animals fast for 12 h and then giving them access to food for an hour. Two hours later they are euthanized. Afterwards, the stomach content is weighed, and the amount of food still left in the stomach and how much was consumed is compared. This will show the animal’s stomach motility [194]. Stool collection is also widely used. This shows colon motility. By placing a mouse in an autoclaved cage as feces are formed. They are collected and stored in a sterile EP tube [195].

Toxin models 6-OHDA lesion rats presented depleted gastric motility, with delayed intestinal transit shown by X-ray imaging [135]. Gastric dysfunctions can be seen in both bilateral [135] and unilateral infusions of 6-OHDA [196].

Although other toxin models also express GI dysfunction, it is only through peripheral administration. As seen in this study, chronic low doses of MPTP were administered twice a week, lasting 5 weeks in total [146]. Another study used paraquat with three weekly injections in rats to assess brain-stem DA effects on gastric motility and tone [163]. Rotenone is no different, with rats that were exposed to five weekly doses for 4 weeks displaying significant delayed gastric emptying, relatively similar to PD patients [158].

The PFF model does not present GI dysfunctions when injected in the striatum. Nevertheless, alterations can be observed when the fibrils are injected directly into the gut [168].

Transgenic models that overexpress an a-synuclein mimic PD patient’s GI dysfunctions well. Additionally, studies use these models to evaluate how the gut–brain axis interacts in PD and possible treatments for the disease [177,197], since the model is very trustworthy.

#### 3.2.3. Anxiety and Depression

Toxin models, 6-OHDA, MPTP, and rotenone, displayed depression-like and anxiety-like behaviors in lesioned rats [136]. Likewise, reserpine also showed depression-like [153] and anxiety-like behavior [154]. However, this was achieved through recurrent low doses. On the other hand, paraquat was only able to produce anxiety-like and depression-like behaviors combined with maneb [164].

Models that use a-synuclein also tend to cause these behaviors, especially PFF models, which cause depression-like and anxiety-like behaviors in mice either with an injection directly to the brain [198] or peripherally through the gut–brain axis [168].

Genetic models are not able to replicate depression-like and anxiety-like behaviors similar to the ones in PD patients. Although some that displayed mildly none were able to mimic what we see in humans completely [19].

In general, the main tool to assess anxiety-like behavior in the PD model is through the open field test, focusing on analyzing the time that the animal stays in the center of the field. As for depression-like behavior, the main tool used is the forced swimming test, consisting of analyzing total immobility time (when the rat makes no active effort to swim), total swimming time, and frequency of escape attempts [136].

Overall, rodents present themselves as a useful animal model for PD, especially due to their capacity to exhibit the PD-like phenotype, having various behavioral tests already established, the possibility of genetic manipulations, and easily available evaluations for non-motor symptoms [199,200,201].

## 4. Predictive Validity

Predictive validity allows us to know if the response seen in experimental animals after their exposure to some kind of intervention, whether a drug, surgical, or other, will be evidenced in humans or if it will reverse behaviors caused by the disease [11].

**Figure 3:**
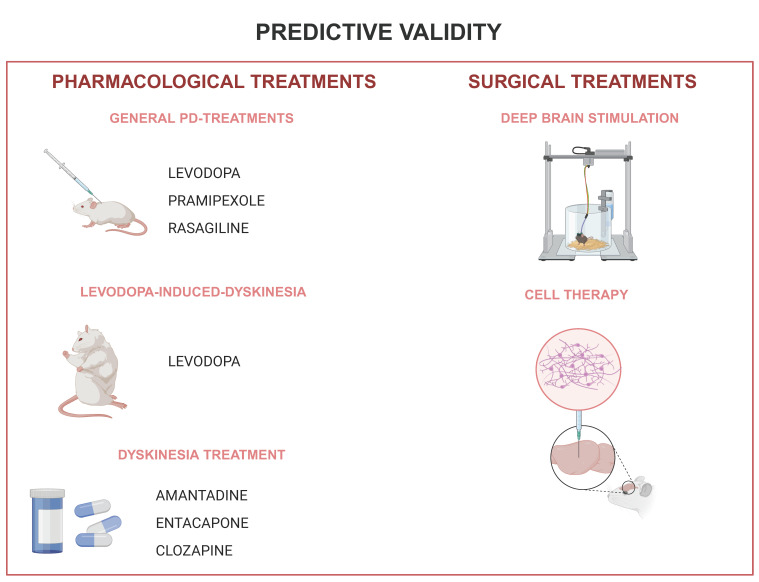
Main treatments used in the predictive validation of rodent models of Parkinson’s disease.

Before assessing this aspect, it is crucial to understand the clinical outcomes in patients who have undergone the intervention. In PD, we have evidence to evaluate the predictive validity related to the therapeutic response of the motor symptoms of the disease or therapeutic progressive complications management, but not yet related to neuroprotection, since we do not have clinically validated therapeutics for this [202]. Summarized information concerning the sections above can be found in Table 3 and Figure 3.

### 4.1. Pharmacological Treatments

Levodopa (L-DOPA) serves as an example of how predictive validity can be established in pharmacological treatments for PD.

Case example: Mr. S., a 55-year-old man, began experiencing intermittent rest tremors in his right hand six months ago. He also noticed slowness of movement, decreased arm swing, and a reduced stride length when walking. As these symptoms worsened, his neurologist diagnosed him with Parkinson’s disease and prescribed L-DOPA at a dose of 300 mg/day, divided into three doses. Following the treatment, Mr. S. experienced significant relief from his motor symptoms; his tremors diminished, his movements became smoother and quicker, and his overall motor function improved substantially.

This improvement highlights the impact of L-DOPA in managing PD symptoms and underscores its status as the gold standard in PD clinical management, providing significant and continuous relief at all stages of the disease [219]. This clinical scenario aligns with findings from rodent models of PD, which have also demonstrated improvements in motor function following L-DOPA administration.

Even though L-DOPA is now widely recognized as the most effective therapy for PD treatment, several years of uncertainty and variable clinical results preceded its introduction, five decades ago. Basic neuroscience research identifying how amino acids cross the blood–brain barrier, as well as defining the role of DA in mammalian brains [219] provided a consistent pathophysiological pathway to drive the initiation of PD treatment with L-DOPA [220] at an initial 300 mg a day dose, reaching over the years doses of 1000 mg to 1500 mg, or even more [203].

The use of L-DOPA in rodent models of PD, when compared to controls, has also shown to be effective in inducing motor performance improvement, even though the protocols used vary a lot regarding the doses applied. Paillé and colleagues (2007) used L-DOPA in rats to reverse severe bradykinetic behavior induced after 6-OHDA injection compared to controls, using a 100 mg/kg dose [205], while other researchers used a 12.5 mg/kg dose [206].

In recent years, other drug treatments for PD management aimed at different pathophysiological targets, one being dopaminergic agonists [221]. In this category of substances, pramipexole has proven efficacy and broad clinical applicability at doses between 0.125 mg and 4.5 mg/day [203,204]. In rodent PD models, this substance reversed akinesia induced by unilateral injection of 6-OHDA at a dose of 0.05 mg/Kg [207], and in the reserpine-induced PD model, at doses of 0.1, 0.3 and 1 mg/Kg [208].

Another class of substances used widely in PD treatment is monoamine oxide inhibitors—B (MAOI—B). Currently, the most used drug in this class is rasagiline, at a dose of 1 mg/day [203]. In the rotenone-induced PD rodent model, a controlled release system developed for rasagiline, delivering the medication at 1 mg/kg/day, effectively reduced akinesia [209].

It is essential to recognize that the translational gap between promising animal studies and effective disease-modifying therapies is a significant challenge in neurodegenerative disease research [222]. This gap is not necessarily due to a lack of validity in animal models, but rather reflects challenges in the structuring of experimental hypotheses. While rodent models have been invaluable in advancing our understanding of disease mechanisms, translating these findings into more complex human systems remains elusive. Rodent studies often prioritize demonstrating therapeutic potential over confirming translational relevance, which contributes to this gap [223].

Despite efforts to develop guidelines aimed at improving the translational potential of preclinical studies [224], this gap becomes particularly evident when promising preclinical results fail to translate into successful Phase II and III clinical trials, where the primary reason for failure is often the inability to demonstrate treatment efficacy [225]. The robustness of efficacy demonstrated in animal models before advancing to human trials is frequently questioned [226]. Convincing preclinical efficacy with high translational potential, reducing potential risks to participants, and conducting an unbiased, critical assessment of the aggregate evidence supporting the overall therapeutic approach could help reduce this translational gap [227]. Additionally, modification in academic reward systems that prioritize discovery over the translation of these findings into practical medical advancements is needed to mitigate these difficulties [228].

### 4.2. Treatment Adverse Effect

Case continuation: During Mr. S.’s clinical follow-up, adjustments were made to his L-DOPA dosage over the first six years. The dose was gradually increased from 300 mg/day to 600 mg/day. Additionally, a dopamine agonist, pramipexole, was introduced, reaching a dose of 0.75 mg/day. After this period, Mr. S. began to experience complications commonly associated with long-term use of the medication. He noticed periods during the day when his motor symptoms re-emerged or worsened, despite taking his medication. These episodes, known as “off” periods, were accompanied by increased stiffness and temporarily decreased gait performance. Additionally, Mr. S. developed involuntary, erratic movements known as dyskinesias, which often appeared when the medication was at its peak effectiveness. These complications significantly impacted his daily quality of life.

After some years of L-DOPA use for the treatment of PD, initially, in some periods of the day, patients present moments called “off state”, in which motor performance decay, sometimes associated with motor block phenomena in lower limbs, progressive shortening of the medication’s effect time, as well as moments in which treatment do not take effect or take too long to start acting [229]. These changes and others in motor performance are called “motor fluctuations”. They seem to arise from the neuronal activity and gene expression changes associated with pulsatile L-DOPA administration loss [230]. Some groups reproduced motor fluctuations in the rodent model of PD [213,231].

Another well-described phenomenon related to long-term treatment evolution is dyskinesias. It can be defined as abnormal and complex involuntary movements with dystonic, choreic, ballistic, or myoclonic patterns, but frequently assuming a combination of them, arising some years after the initiation of L-DOPA treatment. There are three main types of L-DOPA-induced dyskinesias (LIDS): peak-dose dyskinesias, with a predominantly choreiform pattern; diphasic dyskinesias, which intersperse exacerbated involuntary movement with periods of significant bradykinesias; and so-called “off” dystonias, usually identified in the morning after hours without L-DOPA supplementation, usually in the lower limbs [232]. LID’s physiopathology is still not well understood, but preclinical and clinical data show a complex origin involving presynaptic and postsynaptic mechanisms, different DA and ionotropic and metabotropic glutamate receptors, as well as non-dopaminergic neurotransmitter systems [233].

Although the dyskinetic phenomenon associated with L-DOPA is now well defined and present in rodent animal models of PD, there is still no consensus on the dose of L-DOPA used to reproduce this phenomenon, route of administration, or time of exposure to the drug. Research protocols have used a wide dose range varying from 1 mg/kg to 300 mg/kg [210].

The non-human primate MPTP model was the first research platform used to accurately reproduce the motor features of human dyskinesia but is limited by factors such as high costs and non-standardized methodologies [139]. Currently, 6-OHDA intracerebral infusion in rodents is an established research platform for reproducing dyskinesia, offering versatility in terms of injection coordinates and toxin doses, making it suitable for mimicking different stages [234]. Additionally, this model demonstrates drug-induced rotational behaviors [235]. Rotation has become the main behavioral assessment in the unilateral 6-OHDA-lesioned rat model, which was developed in the 1960s. This is mainly due to the lack of other motor impairments present in human PD, such as rigidity and resting tremors [236]. Changes in this rotation behavior started to be used as models for motor fluctuations and a rotation sensitization model was introduced to measure dyskinesia in rats [129]. Previously believed as a motor response to medication, the rotatory behavior displayed by Parkinsonian rodents is now recognized as LIDs, with a pattern more compatible with human peak-dose dyskinesias [237].

To assess dyskinesia in rodents, researchers have developed rating scales and classifications of abnormal involuntary movements (AIMs) [130,131]. To assess rotational motor behavior, apomorphine or amphetamine rotation tests can also be employed [238]. All these tests can also be employed in PFFs-based models. Genetic rodent models of dyskinesia have been developed since the 1990s and are becoming more popular due to their time and cost-effectiveness [170]. The genetic models that are appropriate for dyskinesia research are those that demonstrate a significant decline in nigrostriatal dopaminergic function [239]. They can be analyzed by using the mentioned dyskinesia assessment protocols [240].

Reserpine has also shown oral dyskinesia in rodent models. This motor symptom is usually tested through the measurement of oral movements, such as the number of tongue protrusions (projection of the tongue out of the oral cavity), the length of time that facial muscles twitch, and the chewing movement rate not directed toward any physical material [18,148].

Case continuation: After experiencing worsening motor symptoms and increased dyskinesias, Mr. S. sought further evaluation from his neurologist. He recommended a gradual increase in L-DOPA dosage from 600 mg/day to 900 mg/day and from three doses a day to five, as well as an increase in pramipexole from 0.75 mg/day to 1.5 mg/day. While these adjustments improved motor performance, reducing bradykinesia, tremors, and gait complaints, they also led to a significant increase in dyskinesias. To address these side effects, amantadine was gradually introduced at a dose of 300 mg/day, resulting in improved motor symptoms.

Treating dyskinesia is burdensome, but some drugs are available for clinical management. One example is amantadine, the only drug currently licensed for LIDs treatment [221,241], showing concomitant antiparkinsonian properties (due to its dopaminergic effects), and anti-dyskinetic properties may be due to its multiple action mechanisms involving serotoninergic, noradrenergic, GABAergic, and mainly anti-glutamatergic effects [221].

Amantadine is currently used for the clinical management of dyskinesias in Parkinsonian patients at a dose of 300 mg/day [211]. Amantadine is also used in research protocols using PD rodent models with several therapeutic regimens, ranging from 10 to 60 mg/kg doses, usually as a single dose [212].

In trying to overcome motor fluctuations in PD, strategies to prolong the L-DOPA effect time are frequently used [204]. Likewise, catechol-O-methyltransferase inhibitors (COMTI) act with this effect, decreasing DA degradation in the CNS.

Entacapone, the most widely used COMTI today, currently coupled to L-DOPA intake, is a well-defined clinical strategy for PD motor symptoms and motor fluctuations management. The entacapone doses vary from 600 to 1600 mg/day [222]. In rodents, entacapone associated with L-DOPA in a 30 mg/kg/day dose delayed the onset of dyskinesias and reduced its intensity [213].

Clozapine is also used for PD dyskinesias management (Durif, 2004), and it showed effectiveness in reducing this symptom at a dose of 39.4 + −4.5 mg/day. In the PD rodent model, Lundblad and co-workers (2002) also obtained a favorable response in reducing dyskinesias at an 8 mg/kg dose.

Several other drugs showed antidyskinetic effects in humans and were also successfully tested in this animal model, but currently, with no widespread clinical use. Some of these medications are yohimbine at a dose of 10 mg/kg, naloxone at a dose of 4 ± 8 mg/kg, and 5-methoxy 5-N, N-dimethyl-tryptamine at a 2 mg/kg dose [215].

### 4.3. Surgical Treatments

Case continuation: After three years, despite optimized pharmacological therapy, Mr. S. continued to experience bothersome motor fluctuations and dyskinesias. His neurologist recommended deep brain stimulation (DBS) as a surgical intervention. The procedure involved placing electrodes bilaterally in the subthalamic nucleus. The surgery was performed without complications and resulted in a significant motor improvement that allowed a reduction in medication, which was accompanied by a concurrent improvement in dyskinesias.

Before the widespread use of L-DOPA for PD treatment, ablative surgical procedures by lesioning brain structures to improve patients’ motor performance were widely used. After L-DOPA, many of these procedures fell into disuse, but in selected groups of patients, there is still room for these procedures [242]. It became clear that L-DOPA alone would not be the solution since motor fluctuations and dyskinesias appeared after a few years of medication use. Also, since the 1980s, brain imaging technology, with the diffusion of tomography and nuclear magnetic resonance techniques, has led to greater accuracy in neurosurgical procedures. Thus, surgical treatments gained renewed interest [243], and since the late 1980s and early 1990s, there has been a revolution in the treatment of the advanced stage of PD through a surgical treatment called deep brain stimulation (DBS), delivering electrical impulses into deep brain structures using electrodes installed through stereotaxis, with evident motor improvement [244].

There are consistent results on the favorable motor response after ablative neurosurgical treatments in humans [245], as well as in rodent models of PD [246], and also after DBS in rodent models of PD in both the subthalamic nucleus (STN) and the globus pallidus (GP) or the entopeduncular nucleus (EN) as dominated in rodents [244,247], classical targets aimed in human patients. DBS induces electrophysiological patterns in the PD rodent model that are similar to humans. The STN high-frequency stimulation decreases its neural activity in PD rodent models [248], as well as in PD patients [249].

Well-defined parameters of STN-DBS in humans to improve rigidity, tremors, and bradykinesia comprise monopolar stimulation with voltages above 3 V, frequencies around 130–185 Hz, and pulse widths around 60–90 μs [216]. The frequencies and pulse widths commonly used in animal research are similar to those used in clinical practice [218]. However, the stimulation amplitudes used in experimental PD rodent models are in the order of ten times less intense than in humans in contrast to the current density delivered to the neuronal tissues of rodent models, more than ten times higher, due to the difference in the diameters of the electrodes used in humans (with a diameter of 6 mm^2^) and in rodents (1–100 μm^2^) [217].

Another surgical procedure developed for PD treatment was based on cell therapy, which consists of the implantation of dopaminergic neurons that after being implanted inside the striatum, would start producing dopamine. The first clinical studies were started in the 1990s but only after the early 2000s a complication was identified in patients undergoing this procedure, called transplant-induced dyskinesias [250], which is different from LIDs in having no temporal relationship with taking medication, as well as in the semiology of the phenomenon [251].

The PD mouse model exposed to 6-OHDA is the only one that can reproduce dyskinesias induced by the implantation of dopaminergic cells, and its use allowed exploring factors that may be decisive for the appearance of this phenomenon, such as the site of the implant, its size and the type of cells in it, the previous presence of LIDs, and the host inflammatory response to the implant [250].

## 5. Future Perspectives

This paper offers a detailed description of how the main hallmarks of PD can be validated in animal models using rodents, intending to provide researchers with a useful tool to help them choose the ideal animal model for their research. Unlike other reviews on animal models of PD, our article does not focus on just describing the animal models. Instead, we focus on the characteristics of the disease, showing how these characteristics are validated experimentally. We also emphasize the importance of verifying and following the principles of the three types of validations when creating new animal models in the context of the translational study of PD, considering that most new treatments fail clinical trials.

When it comes to translational research into PD, it is important to note that although the neuroanatomy of rodents is similar to that of humans, it is not identical, neither does spontaneous neurodegeneration occur in rodent brains, which in turn makes a huge difference when comparing the testing of new treatments in animal models and humans. In addition, there is no ideal and identical protocol used by all researchers to induce the models, differing mainly in the protocol, doses used, and target structures in the brain. Therefore, although many models have all three types of validation, as shown in Table 1, it is important to point out that only the form of induction is the same and that the results often differ in terms of protocol, dose, and target, as mentioned above, which means that the validations refer much more to the form of induction of the model than to the animal model itself.

Even with all the variations of existing protocols, there is no ideal model that can be used in all research. First, the complete understanding of the pathophysiological mechanisms of PD is still not fully elucidated. Current models aim to address the two most evident characteristics we now have. Therefore, an ideal model would need to primarily simulate the progression of dopaminergic neuron degeneration and the presence of a-synuclein aggregates, ensuring that both characteristics are highly evident. However, it is very challenging to find a single animal model using the same protocol that can adequately represent both features, even though these characteristics can be simulated in individual animal models with different protocols for each.

Since no individual animal model can concretely simulate these two characteristics, a promising alternative would be the combination of animal models with the distinct features of each. This approach has already been initiated by a few research groups and can be referred to as combined models or “fusion models” [10]. These models are primarily based on a combination of genetic models with neurotoxins. Some of the studies conducted so far show that the overexpression of a-synuclein combined with the infusion of MPTP appears to exacerbate the injury [252,253], while the overexpression of a-synuclein appeared to have an effect of neuroprotection in the neurons following paraquat infusion [185,254,255,256], and the same effect was demonstrated following MPTP infusion in a-synuclein -deficient models. Also, it is important to explore other types of combinations, as some groups have explored combining models based on AAV-aSyn with a-synuclein PFFs [257,258], showing that the combined model can increase injury when compared to each of the models individually [259].

In conclusion, research focused on elucidating the relationship between a-synuclein aggregates and dopaminergic neuron loss within the same animal model is essential. The creation of combined models stands out as an excellent strategy for testing new drugs before clinical trials. Furthermore, developing these models is crucial not only for the search for new therapies but also for understanding the pathophysiological mechanisms of PD and how these factors interrelate. This approach has the potential to significantly advance our knowledge and treatment of PD.

## Figures and Tables

**Table 1:** Summary of DA neuronal loss and aSyn aggregation on construct validation of animal models of Parkinson’s disease in rodents.

Model	Da Neurons Loss	Asyn-Aggregation
6-OHDA	Yes [7,12,13,14]	No [15]
MPTP	Yes [16]	No
RESERPINE	Yes [17,18]	Yes [19]
ROTENONE	Yes [20,21,22]	Yes [20,21,22]
PARAQUAT	Yes [23,24,25]	Yes [26,27]
PFFs	Yes [28,29,30]	Yes [28,29,30]
AAV-aSYN	Yes [31,32]	Yes [31,32]
TRANSGENIC	Yes [33]	Yes [33,34,35,36]

**Table 2:** Overview of the main rodent models of Parkinson’s disease, their described motor and non-motor symptoms, and the tests used to confirm face validity.

Model	Motor Symptoms	Tests for Motor Symptoms	Non-Motor Symptoms	Tests for Non-Motor Symptoms
6-OHDA	Dysfunctions in locomotor activities (akinesia, bradykinesia, hypokinesia), muscle rigidity [123,124], dyskinesia, and gait abnormalities [90].	Open field, rotarod, pole test [125], tail suspension test (TST), tail suspension swing test (TSST) [126], Catwalk [127,128], rotational sensitization [129], grasping test or movement-induced reflex electromyographic activity [123,124], AIMs and other rating scales for dyskinesia [130,131], rotational motor behavior (apomorphine or amphetamine tests) [132], and cylinder test [133].	Sleep abnormalities [134] Gastrointestinal dysfunction [135] Anxiety and depression [136]	EEG Gastric emptying [135] Sucrose preference test Forced swimming test Open field test [136]
MPTP	Dysfunctions in locomotor activities (akinesia, bradykinesia, hypokinesia), dyskinesia, resting tremors, muscle rigidity, and gait abnormalities [137,138,139].	Open field, rotarod, balance beam test [140] stepping test [141], pole test [142], horizontal and vertical grid tests [143], swim-test [144], catalepsy test, cylinder test, Catwalk [128].	Sleep abnormalities [145] Gastrointestinal dysfunction [146] Anxiety and depression [136]	EEG [145] Stool Collection [146] Sucrose preference test Forced swimming test Open field test [136]
RESERPINE	Akinesia [147], oral dyskinesia [148,149], and muscle rigidity.	Open field, rotarod, catalepsy test, and oral dyskinesia assessment [148,150,151].	Sleep abnormalities [152] Anxiety [153] depression [154]	EEG [152] Open field Elevated plus maze [154],
ROTENONE	Dysfunctions in locomotor activities (akinesia, bradykinesia, hypokinesia), muscle rigidity [155], gait abnormalities [156].	Open field, rotarod, pole test, forced swimming test [157], catalepsy test, cylinder test.	Gastrointestinal dysfunction [158] Anxiety and depression [136]	Stool collection [159] Urine collection [159] Gastric emptying [158] Bead latency [160] Sucrose preference test Forced swimming test Open field test [136]
PARAQUAT	Dysfunctions in locomotor activities (akinesia, bradykinesia, hypokinesia) [161], and forelimb rigidity [162].	Open field, rotarod, inclined plane test, swimming test, catalepsy test, wire suspension test [161].	Gastrointestinal dysfunction [163] Anxiety and depression [164]	Gastric motility [163] Sucrose preference test Forced swimming test Open field test Elevated plus maze [164]
PFFs	Dysfunctions in locomotor activities (akinesia, bradykinesia, hypokinesia) [28,147], dyskinesia.	Open field, rotarod, wire hang test, tail suspension test (TST) [28], muscular strength test, elevated plus maze test, forced swim test, Y-maze test, apomorphine-induced rotational behavior test [165].	Sleep abnormalities [166] Gastrointestinal dysfunction [167] Anxiety and depression [168]	EEG [166] Stool collection [167] Open field Elevated plus maze Tail suspension [168]
GENETIC	Dysfunctions in locomotor and sensorimotor activities (akinesia, bradykinesia, hypokinesia) [169], dyskinesia [170], and gait abnormalities [171].	Open field, rotarod, tapered balance beam test [171], pole test, adhesive removal test [172], grip strength [173], tail suspension test [174], AIM scores [170], cylinder test [175], ink test [98].	Sleep Abnormalities [176] Gastrointestinal dysfunction [177]	EEG [176] Stool collection [177]

**Table 3:** Summary of the primary treatments used in the predictive validation of rodent models of Parkinson’s disease.

Treatment	Humans	Rodents
Pharmacological Treatment		
Levodopa	300–1500 mg/day [203,204]	12.5–100 mg/kg [205,206]
Pramipexole	0.125–4.5 mg/day [203,204]	0.05–1 mg/kg [207,208]
Rasagiline	1 mg/day [203]	1 mg/kg [209]
Dyskinesia		
Levodopa	-	1–300 mg/kg [210]
Dyskinesia Treatment		
Amantadine	300 mg/day [211]	10–60 mg/kg [212]
Entacapone	600–1600 mg/day [203]	30 mg/kg/day [213]
Clozapine	39.4 + −4.5 mg/day [214]	8 mg/kg dose [215]
Surgical TreatmENT		
DBS	130–185 Hz; 60–90 μs pulses [216], eletrodes size: 6 mm^2^ [217]	130–185 Hz; 60–90 μs pulses [218], eletrodes size: 1–100 μm^2^ [217].

## Data Availability

Data will be made available on request.
